# Association between Three Therapeutic Strategies and Clinical Outcomes of 2009 FIGO Stage IB2/IIA2 Cervical Cancer

**DOI:** 10.1155/2022/9497798

**Published:** 2022-08-22

**Authors:** Yong Zhang, Xiaobi Tang, Shanshan Ma, Meizhu Shen, Li Jiang, Wenchao Yuan, Rensheng Wang, Fang Wu

**Affiliations:** ^1^Department of Radiation Oncology, First Affiliated Hospital of Guangxi Medical University, Nanning, Guangxi, China; ^2^Department of Radiation Oncology, Liuzhou People's Hospital, Liuzhou, Guangxi, China

## Abstract

**Objective:**

The aim of this study was to compare clinical outcomes of three therapeutic strategies in patients with stage IB2/IIA2 cervical cancer.

**Methods:**

This is a retrospective cohort study. Patients diagnosed with stage IB2/IIA2 cervical cancer between April 2010 and December 2015 at First Affiliated Hospital of Guangxi Medical University were included and classed into three groups. The primary outcomes were overall survival (OS) and progression-free survival (PFS). The secondary outcomes included toxicity, hospitalization costs, clinical value, and length of stay.

**Results:**

206 patients were included: 104 used primary surgical treatment (PST), 53 used neoadjuvant chemotherapy followed by radical surgery (NAC + RS), and 49 used concurrent chemoradiotherapy (CCRT). Fewer patients with NAC + RS had deep cervical stromal invasion than primary surgical treatment (PST) (*P*=0.024). 70.2% of PST and 77.4% in NAC + RS required postoperative radiotherapy or chemoradiotherapy (*P*=0.634). Median follow-up was 57 months and the 3-year OS and PFS in PST, NAC + RS, and CCRT group were 87.5%, 84.9%, 85.7% and 85.6%, 79.2%, 85.7% (*P*=0.856 and *P*=0.424, respectively). Three therapeutic strategies were not associated with OS and PFS. Hospitalization costs were significantly higher in NAC + RS compared to PST (*P* < 0.001) and CCRT (*P* < 0.001). Length of stay in NAC + RS was longer than PST (*P* < 0.001) and CCRT group (*P*=0.07).

**Conclusion:**

The results of this study tend to suggest that the three therapeutic strategies were equivalent treatment options for patients with 2009 FIGO stage IB2/IIA2 cervical cancer. However, prospective larger studies are needed to confirm this. In addition, we did find that concurrent chemoradiotherapy needed shorter treatment time and less cost.

## 1. Introduction

Cervical cancer is a common malignant tumor affecting the health of females globally [1]. Concurrent chemoradiotherapy has been the standard treatment of locally advanced cervical cancer (LACC) since 1999 [2–6]. However, 25% to 40% of patients still experience relapse, with a subset experiencing distant failure despite local control after chemoradiation [7]. Patients with tumors measuring >4 cm at the largest diameter have a worse prognosis compared to those with smaller tumors, regardless of treatment [8, 9]. Additionally, the optimal therapeutic strategies for patients with stage IB2/IIA2 cervical cancer remain controversial. Since the 1980s, neoadjuvant chemotherapy (NAC) followed by radical surgery (RS) or concurrent chemoradiotherapy (CCRT) has been proposed and utilized for LACC [10–12]. The rationale for this approach is that it reduces tumor volume, kills subclinical lesions, increases tumor resectability, and eliminates micrometastases. Additionally, chemotherapy given in the neoadjuvant setting might be more effective, partly because it is delivered to uncompromised tumor blood supply and to a population of chemosensitive tumor cells.

Based on several studies, patients have shown significant benefit of NAC + RS over radiotherapy alone or with concurrent chemotherapy in terms of overall survival (OS) and disease-free survival (DFS). Consequently, NAC has emerged as an effective alternative treatment option [13, 14]. Conversely, there have been studies reporting dissimilar findings vis-a-vis the clinical outcomes of NAC [15, 16].

Therefore, the aim of this study was to compare the efficacy, toxicity, hospitalization cost, and length of stay of three therapeutic strategies which were primary surgical treatment (PST), neoadjuvant chemotherapy followed by radical surgery (NAC + RS), and concurrent chemoradiotherapy (CCRT) for patients with stage IB2/IIA2 (based upon International Federation of Gynecology and Obstetrics staging system 2009, FIGO) cervical cancer, to provide evidence for selection of optimal clinical treatment.

## 2. Methods

### 2.1. Study Design and Subjects

We performed a retrospective cohort study of women with 2009 FIGO stage IB2/IIA2 cervical cancer treated by primary surgical treatment (PST), or neoadjuvant chemotherapy followed by radical surgery (NAC + RS), or concurrent chemoradiotherapy (CCRT) in the First Affiliated Hospital of Guangxi Medical University between April 2010 and December 2015. Inclusion criteria were newly diagnosed patients between 18 and 70 years of age with carcinoma of the uterine cervix confirmed via histopathology; Eastern Cooperative Oncology Group (ECOG) performance status of 1 or less; adequate bone marrow function (WBC >3000/mm^3^, platelets >120,000/mm^3^), adequate renal function (blood urea nitrogen <25 mg/dl, creatinine <1.5 mg/dl), and normal liver function (bilirubin <2 mg/dl).The exclusion criteria included the presence of secondary cancers; uncontrollable diabetes, hypertension, or heart disease; pregnant or lactating women; and liver or renal failure.

Patients who were treated with PST received type III radical hysterectomy plus bilateral pelvic lymphadenectomy within 1 to 2 weeks of diagnosis. Patients who were treated with NAC + RS underwent preoperative intravenous platinum-based combination chemotherapy, consisting of paclitaxel 135 mg/m^2^ plus cisplatin 75 mg/m^2^·day once every 3 weeks for 2 to 3 cycles, and received type III radical hysterectomy plus bilateral pelvic lymphadenectomy 3 to 4 weeks after the last course of chemotherapy. In the PST and NAC + RS groups, postoperative adjuvant therapy (radiotherapy or concomitant chemotherapy and radiotherapy) was administered in accordance with published evidence [2, 17]. On the basis of histopathologic evaluation of the surgical specimen, adjuvant chemoradiation was given in the presence of any one of the following features: lymph node metastasis, positive vaginal margins, and parametrial involvement. Adjuvant radiotherapy alone was given based on the presence of any two of the following features: deep cervical stromal invasion, lymphovascular invasion, or tumor size ＞4 cm.

Patients treated with CCRT received external-beam radiation (EBRT) to the whole pelvis and brachytherapy. EBRT was delivered at a dose of 45∼50 Gy in 25 fractions (1.8∼2.0 Gy per fraction, using IMRT technique), followed by intracavitary radiation at a dose of 28 Gy in 4 fractions to point A twice a week. Patients were also given cisplatin at a dose of 40 mg/m^2^ once a week continuously for 4 to 5 weeks.

As antiemetic agents, the combination of a steroid and ondansetron hydrochloride or granisetron hydrochloride was administered before chemotherapy. Prophylactic use of recombinant granulocyte colony-stimulating factor was not allowed.

The study was approved by the Medical Ethics Committee of First Affiliated Hospital of Guangxi Medical University. Informed consent was exempted from the study.

### 2.2. Data Collection and Follow-Up

Demographic data and clinical variables collected included age, pretreatment hemoglobin, FIGO stage, differentiation degree, histology type, tumor size, lymph node metastasis, vascular tumor thrombus, vaginal margin, deep stromal invasion, and any adjuvant treatment of patients.

All patients were evaluated once a week during treatment. The first assessment of tumor response was performed 3 months after the completion of treatment by physical examination, pelvic magnetic resonance imaging (MRI), or computed tomography (CT) with contrast and chest/abdomen CT with contrast. Then patients were follow up every 3 months during the first 2 years, every 6 months from 3 to 5 years, and then annually. All patients were followed up via medical record or phone interviews to vital status December 31, 2018.

Acute and late treatment-related morbidities were assessed according to the Common Terminology Criteria for Adverse Events (CTCAE) version 5.0. Physical examination, chest CT, abdomen ultrasound, and laboratory analysis were performed at each follow-up. Pelvic MRI or CT was performed every 6 months after the first assessment of tumor response. Cervical/vaginal cytology test was performed annually. For patients with suspicious recurrent/metastatic disease, additional imaging such as positron emission tomography (PET-CT), bone scintigraphy, or a biopsy whenever possible was obtained to confirm.

In addition, the surgical approach, length of hospital stay, and medical costs were also documented. Length of stay (LOS) was defined as the number of inpatient days from the time of admission to the time of discharge from the hospital. The direct medical cost was estimated as the total expenditure during the hospital stay, including hospitalization expenses, cost of treatments, and cost of examinations.

### 2.3. Outcomes

The primary end point was overall survival (OS) calculated from the date of entry into the study to the date of death or the last follow-up visit and progression-free survival (PFS) defined as the time between entry into the study and progression of the tumor (in any respect) or death (from any cause). The secondary outcomes included toxicity, hospitalization costs, clinical value, and length of stay.

### 2.4. Statistical Analysis

Data was presented as mean ± standard deviation (SD) for continuous variables conforming to normal distribution and median with a range was used for nonnormal distributions after normality testing. Frequency and proportion were used for categorical variables. Statistical comparisons of intergroup differences in characteristics and postoperative adjuvant therapy rate, as well as the incidence of acute and late toxicity, were performed using the Chi-square test or Fisher exact test. LOS and treatment costs among the three groups were compared by analysis of variance. Survival was analyzed using the Kaplan–Meier method and log-rank test. Univariate analysis was performed using the log-rank test to identify parameters associated with treatment outcomes. Univariate analyses were performed to identify which factors affected patient outcomes and to evaluate the prognostic importance of age, pretreatment hemoglobin, differentiation degree, histology type, stage, tumor size, therapeutic regimen, lymph node metastasis, vascular tumor thrombus, vaginal margin, and deep stromal invasion. Multivariate Cox-regression analyses were conducted to identify independent prognostic factors. All data were analyzed using the SPSS Statistics 22.0 (IBM Corp., Armonk, NY, USA). *P* < 0.05 was construed as statistically significant.

## 3. Results

Of the 206 patients who were enrolled, 126 (55.6% of the PST group, 26.2% of the NAC + RS group, and 18.2% of the CCRT group) had 2009 FIGO stage IB2 and 80 (42.5% of the PST group, 25.0% of the NAC + RS group, and 32.5% of the CCRT group) had stage IIA2 with the mean age being 46 years (range 22–69). Pathological type distributions were as follows: squamous carcinoma, 174; and nonsquamous carcinoma, 32. Of all the patients, 104, 53, and 49 cases were classified into the PST, NAC + RS, and CCRT groups, respectively. Clinical characteristics are depicted in Table 1.

Twenty-three patients (22.1%), 2 (1.9%), 14 (13.5%), and 79 (76.0%) in the PST group had lymph node metastasis, positive vaginal margin, vascular tumor thrombus, and deep stromal invasion. Thirteen patients (24.5%), 2 (3.8%), 6 (11.3%), and 31 (58.5%) in the NAC + RS group had lymph node metastasis, positive vaginal margin, vascular tumor thrombus, and deep stromal invasion. No patients in the two groups had parametrial involvement. Because surgery was not performed in patients in the CCRT group and further lymph node metastasis, vaginal margin, vascular tumor thrombus, and deep stromal invasion were not confirmed by pathology, the CCRT group was not added to our statistical analysis in terms of lymph node metastasis, vaginal margin, vascular tumor thrombus, and deep stromal invasion.

Fewer patients in NAC + RS group had deep cervical stromal invasion than those in PST group (*P*=0.024). No differences were observed in lymph node metastasis, vascular tumor thrombus, and vaginal margins. The rate of postoperative adjuvant treatment between PST and NAC + RS group was 70.2% in PST group compared with 77.4% in NAC + RS group who required postoperative treatment. Of those, 26.9% compared with 30.2%needed radiotherapy only and those undergoing chemoradiotherapy was 43.3% compared with 47.2% (Table 2).

**Figure 1:**
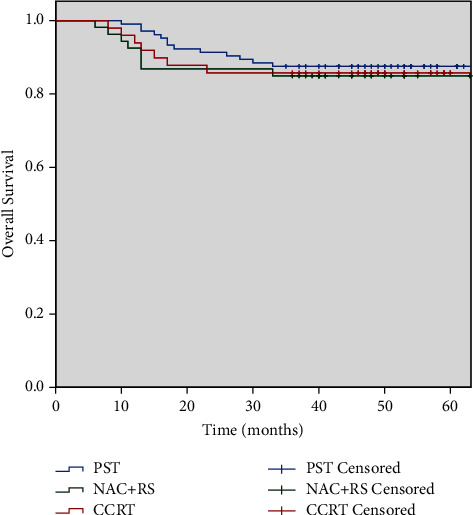
Overall survival comparison between three groups (*P*=0.856). PST, primary surgical treatment; NAC + RS, neoadjuvant chemotherapy followed by radical surgery; CCRT, concurrent chemoradiotherapy.

The median follow-up was 57 months (range 4–104 months) during which time 28 of 206 patients died: 13 in PST group, 8 in NAC + RS, and 7 in CCRT. The median time of death of these 28 patients was 14 months (range 6–33 months). For all patients, the 3-year OS was 86.4%. No significant difference was found among the three groups (Figure 1).

**Figure 2:**
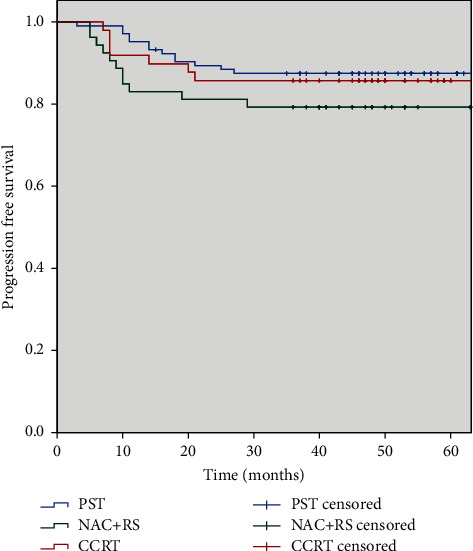
Progression-free survival comparison between three groups (*P*=0.424). PST, primary surgical treatment; NAC + RS, neoadjuvant chemotherapy followed by radical surgery; CCRT, concurrent chemoradiotherapy.

32 of 206 patients were treatment failures. Local recurrence was observed in 13 patients (4 in PST, 7 in NAC + RS, and 2 in CCRT, respectively) after a median time of 14 months (range 10–33 months) in PST, 12.5 months (range 6–29 months) in NAC + RS, 13 months (range 8–15 months) in CCRT. 12 patients experienced distant metastasis (6 in PST, 3 in NAC + RS and 3 in CCRT) after a median time of 14 months (range 10–27 months) in PST, 13 months (range 6–29 months) in NAC + RS, and 15 months (range 7–21 months) in CCRT. Both local recurrence and distant metastasis were observed in 7 patients (4 in PST, 1 in NAC + RS, and 2 in CCRT) after a median time of 12 months (range 10–14 months), 19 months, and 13 months (range 8–15 months), respectively. For all patients, the 3-year PFS rates were 84.0% with no significant differences among the three groups (PST: 85.6%, NAC + RS: 79.2%, CCRT: 85.7%, *P*=0.424) (Figure 2).

In univariate analyses, no significant variables in OS and PFS were found (Table 3). Confounding factors were examined by Cox regression analysis and no significant variables in OS and PFS were revealed (Table 4).

The most frequently observed acute toxicities were hematologic side effects. 11 patients (11.5%) in PST, 12 (27.9%) in NAC + RS, and 17 (39.5%) in CCRT experienced grade 3-4 leukopenia (*P*=0.248). 10 patients (10.4%) in PST, 11 (25.6%) in NAC + RS, and 12 (27.9%) in CCRT underwent grade 3-4 neutropenia (*P*=0.435). 3 (3.1%) in PST, 1 (2.3%) in NAC + RS, and 1 (2.3%) in CCRT suffered grade 3-4 thrombocytopenia (*P*=0.116). 4 (4.2%) in PST, 11 (25.6%) in NAC + RS, and 6 (14.0%) in CCRT had grade 3-4 anemia (*P*=0.190). No grade 3-4 hepatotoxicity and nephrotoxic were reported. Detailed data is shown in Table 5. Grade 3-4 late toxicity consisted primarily of lower extremity lymphedema (3.8%), bowel obstruction (2.9%), and thrombosis (1.0%) in PST; lower extremity lymphedema (9.4%) and bowel obstruction (1.9%) in NAC + RS; and radiation-induced rectitis (8.2%) and femoral head necrosis (2.0%) in CCRT. No grade 5 toxicities were observed. There was no significant difference in cumulative late adverse effects rate among the three groups.

Length of stay in NAC + RS was significantly longer than that in PST (*P* < 0.001) but not in CCRT (*P*=0.07). No significant difference in LOS was observed between PST and CCRT (*P*=0.114). Hospitalization costs in NAC + RS were higher than those in PST (*P* < 0.001) and CCRT (*P* < 0.001) (Table 6).

## 4. Discussion

In the present study, three therapeutic strategies were not associated with better outcomes.

The optimal management for patients with stage IB2/IIA2 cervical cancer (2009 FIGO stage) remains controversial and ambiguous. While platin based chemoradiation (CRT) has been the standard treatment for patients with locally advanced cervical cancer since 1999 [2–6], patients with tumors measuring >4 cm in the greatest diameter continue to have a worse prognosis. Chen et al. reported that 142 patients with locally advanced cervical cancer (stage by surgery or to have primary surgery directly [18]. Significantly reduced pelvic lymph node metastasis and parametrial infiltration rates were detected in the NAC group when compared with the primary surgery group (25.0% vs. 42.9%, *P*=0.025; 25.0% vs. 41.4%, *P*=0.038, respectively). A randomized multicenter study from Yang et al. that sought to evaluate the toxicity and curative effect of NAC for stages IB2, IIA2, and IIB cervical cancer found that deep stromal invasion and lymphovascular space invasion (LVSI) were significantly less in the NAC group compared to the DS (directly to primary surgery) group (*P*=0.002), but there was no difference in lymph node metastasis (*P*=0.698) or positive parametrial involvement (*P*=0.469). The rate of postoperative radiotherapy in the NAC group was lower than that of the DS group, although the difference was not significant (58.9% vs. 63.3%, *P*=0.472) [19].

In our present study, the rate of deep stromal invasion in NAC group was significantly lower than that in the PST group, while no significant difference was detected in lymph node metastasis between the two groups. This finding is consistent with the literature and suggests that NAC is effective in reducing risk factors associated with recurrence [19]. A meta-analysis by Kim et al. showed that the use of NAC in FIGO stage IB1-IIA cervical cancer decreased the incidence of risk factors such as large tumor size (≥4 cm) and lymph node metastasis when compared to radical surgery [20]. As a result, NAC reduced the need for adjuvant radiotherapy. In our study, although the NAC group was superior to the PST group in terms of deep stromal invasion, the two groups did not differ significantly with respect to other risk factors. Additionally, we observed no intergroup differences in the rates of adjuvant radiotherapy and chemotherapy which might be due to lack of uniform standards used to determine the need for adjuvant treatment. Additionally, a local tumor with a largest diameter >4 cm is itself a risk factor for recurrence. Therefore, the gynecologic oncologist may still have opted for adjuvant therapy even in the absence of postoperative high-risk factors.

In previous reports, the effect of NAC on improving survival has remained controversial. A meta-analysis showed that NAC plus radical surgery significantly improved OS and decreased local and distant recurrence rates when compared to radical surgery alone in select patients with locally advanced cervical cancer (FIGO stage IB2-IIB) [13]. However, the relationship between NAC and longer DFS could not be demonstrated, and further research is urgently needed to confirm it. Gupta et al. research suggested that the 5-year DFS in patients with stages IB2, IIA, or IIB cervical cancer in the neoadjuvant chemotherapy plus surgery group was 69.3% compared with 76.7% in the concomitant chemoradiation group (*P*=0.038), whereas the corresponding 5-year OS rates were 75.4% and 74.7%, respectively (*P*=0.87) [14]. A retrospective cohort study from Yan et al. grouped the patients with cervical carcinoma stage IB2 or IIA2 according to whether they received NAC or not prior to surgery after which they evaluated the treatment and prognosis of NAC [15]. The results showed that NAC did not significantly affect DFS (*P*=0.453) and OS (*P*=0.933) between the 2 groups. In a multicenter retrospective study, Zhao et al. reported no significant difference in 5-year cumulative survival rate between the NAC and PST groups (83.3% versus 87.2%, *P*=0.418) [16].

A meta-analysis by Marchetti et al. showed severe acute toxicity in the CCRT group compared with neoadjuvant chemotherapy followed by surgery (NAC + S) in stages IB2, IIA and IIB cervical cancer (1994 FIGO stage) [21]. In the study we present here, the main acute adverse effect was hematology toxicity though no significant differences in the three groups were observed. The inconsistency in results with other studies may be attributed to different chemotherapy regimens. Accumulative incidences of grade 3-4 late adverse effects in the three groups were not high and the intergroup differences were not significant. Impact on long term toxicity and quality of life remains to be determined.

Several retrospective analyses suggest that a prolonged RT treatment duration has an adverse effect on outcomes [22–25]. Extending the overall treatment beyond 6 to 8 weeks can result in approximately a 0.5% to 1% decrease in pelvic control and decrease specific survival for each extra day of overall treatment time. Thus, it is generally accepted that the entire RT course (including both EBRT and brachytherapy components) should be completed in a timely fashion, i.e., within 8 weeks. In the study presented here, the average overall treatment time in CCRT was less than 8 weeks. Although we found no impact of treatment time of NAC followed by RS on tumor control, we did find that NAC + RS prolonged the treatment time and increased the hospitalization costs more than that in PST or CCRT groups. Park et al. found no differences in the oncologic outcomes between CCRT and radical hysterectomy followed by tailored adjuvant therapy in patients with early cervical cancer; however, 88.7% of patients required adjuvant radiotherapy after surgery. These findings suggest that CCRT can avoid unplanned trimodality therapy without compromising oncologic outcomes [26]. In the present study, 70.2% of patients in the PST group and 77.4% in the NAC + RS group required postoperative radiotherapy or chemoradiotherapy, which need the patients return multiple times to the hospital for treatment. This may be one of the reasons for the prolonged treatment time and increased hospitalization costs in the NAC + RS group.

One limitation of our study is that it is a small sample size single-center retrospective study and the follow-up period brief. Given that the study was retrospective, we were constrained by data available in medical records and, in consequence, no analysis was performed regarding a response rate, which is the most vital criterion for improvement of curative effects. Additionally, quality of life was not retrospectively measured. Further randomized trials are needed to explore clinical outcomes in patients undergoing different treatment modalities.

## 5. Conclusions

The results of this study tend to suggest that the three therapeutic strategies were equivalent treatment options for patients with 2009 FIGO stage IB2/IIA2 cervical cancer. However, prospective larger studies are needed to confirm this. In addition, we did find that concurrent chemoradiotherapy needs shorter treatment time and less cost.

## Figures and Tables

**Table 1:** Patient characteristics.

Characteristics	PST *n* (%)	NAC + RS *n* (%)	CCRT *n* (%)	*P*
Age (years)				
>45	54 (51.9)	27 (50.9)	34 (69.4)	0.090
≤45	50 (48.1)	26 (49.1)	15 (30.6)	

Pretreatment hemoglobin				
≥115 g/L	52 (50.0)	25 (47.2)	33 (67.3)	0.077
＜115 g/L	52 (50.0)	28 (52.8)	16 (32.7)	

FIGO stage				
IB2	70 (67.3)	33 (62.3)	23 (46.9)	0.054
IIA2	34 (32.7)	20 (37.7)	26 (53.1)	

Differentiation degree				
Grade 1	4 (3.8)	1 (1.9)	0 (0)	0.357
Grade 2	72 (69.2)	40 (75.5)	39 (79.6)	
Grade 3	28 (27.0)	12 (22.6)	10 (20.4)	

Histology type				
SCC	82 (78.8)	47 (88.7)	45 (91.8)	0.072
Non-SCC	22 (21.2)	6 (11.3)	4 (8.2)	

Tumor size				
>4 cm	51 (49.0)	26 (49.1)	22 (44.9)	0.085
>5 cm	46 (44.2)	20 (37.7)	16 (32.7)	
>6 cm	7 (6.8)	7 (13.2)	11 (22.4)	

**Table 2:** Postoperative RFs associated with pathology.

Characteristics	PST *n* (%)	NAC + RS *n* (%)	*P*
Lymph node metastasis			
Negative	81 (77.9)	40 (75.5)	0.734
Positive	23 (22.1)	13 (24.5)	

Vascular tumor thrombus			
No	90 (86.5)	47 (88.7)	0.704
Yes	14 (13.5)	6 (11.3)	

Vaginal margin			
Negative	102 (98.1)	51 (96.2)	0.487
Positive	2 (1.9)	2 (3.8)	

Deep stromal invasion			
No	25 (24.0)	22 (41.5)	0.024
Yes	79 (76.0)	31 (58.5)	

Adjuvant treatment			
Nontreatment	31 (29.8)	12 (22.6)	0.634
Radiotherapy	28 (26.9)	16 (30.2)	
Chemoradiotherapy	45 (43.3)	25 (47.2)	

**Table 3:** Effect of prognostic factors on survival in univariate analyses.

Factors	*n*	3 y OS (%)	*P*	3 y PFS (%)	*P*
Age (years)					
>45	114	88.6	0.311	86.0	0.431
≤45	92	83.7		81.5	

Pretreatment hemoglobin					
≥115	110	87.3	0731	86.4	0.362
＜115	96	84.9		81.34	

Differentiation degree					
G1-2	156	89.1	0.05	85.9	0.238
G3	50	78.0		78.0	

Histology type					
SCC	174	87.4	0.384	84.5	0.736
Non-SCC	32	81.3		81.3	

Stage					
IB2	126	86.5	0.982	84.1	0.914
IIA2	80	86.3		83.8	

Tumor size					
>4 cm	100	88.0	0.786	87.0	0.471
>5 cm	81	85.2		81.5	
>6 cm	25	84.0		80.0	

Therapeutic regimen					
PST	104	87.5	0.856	85.6	0.424
NAC + RS	53	84.9		79.2	
CCRT	49	85.7		85.7	

Lymph node metastasis^#^					
Yes	36	86.1	0.968	82.3	0.964
No	121	86.8		83.5	

Vascular tumor thrombus^#^					
Yes	20	80.0	0.428	80.0	0.738
No	137	87.6		83.9	

Vaginal margin^#^					
Yes	4	75.0	0.396	75.0	0.545
No	153	86.9		83.7	

Deep stromal invasion^#^					
Yes	105	81.8	0.157	81.0	0.280
No	52	92.3		88.5	

^#^For lymph node metastasis, vascular tumor thrombus, vaginal margin, and deep stromal invasion, only 157 patients in the PST and NAC + RS groups were included in the statistical analysis.

**Table 4:** Multivariate survival analysis.

	OS			PFS		
	HR	95%CI	*P*	HR	95%CI	*P*
Age (years)	0.759	0.808–5.646	0.126	0.509	0.710–3.900	0.241
Pretreatment hemoglobin	−0.002	0.404–2.464	0.997	0.284	0.282–1.713	0.500
Differentiation degree	−0.708	0.186–1.307	0.155	−0.363	0.405–3.373	0.429
Histology type	−0.107	0.295–2.735	0.851	0.156	0.327–2.050	0.773
FIGO stage	−0.203	0.297–2.245	0.694	−0.200	0.126–1.451	0.669
Tumor size	−0.574	0.142–2.236	0.415	−0.849	0.180–2.076	0.173
Lymph node metastasis	−0.081	0.320–2.657	0.881	0.010	0.387–2.634	0.984
Vascular tumor thrombus	−0.506	0.182–1.998	0.408	−0.289	0.240–2.341	0.619
Vaginal margin	−1.220	0.030–2.879	0.294	−0.477	0.069–5.594	0.671
Deep stromal invasion	−0.664	0.163–1.623	0.257	−0.514	0.227–1.575	0.298
Adjuvant treatment	−0.048	0.327–2.778	0.929	−0.287	0.311–1.813	0.524

Therapeutic strategies						
PST	Reference					
NAC + RS	1.28	0.53–3.088	0.583	1.559	0.716–3.394	0.264
CCRT	1.192	0.476–2.988	0.708	1.012	0.413–2.482	0.979

**Table 5:** Frequency of acute and late toxicities.

Toxicity	PST (*n* = 104) *n* (%)		NAC + RS (*n* = 53) *n* (%)		CCRT (*n* = 49) *n* (%)		*P*
	G1-2	G3-4	G1-2	G3-4	G1-2	G3-4	—
Leukopenia	30 (28.8)	11 (10.6)	17 (32.1)	12 (22.6)	22 (44.9)	17 (34.7)	0.248
Neutropenia	21 (20.2)	10 (9.6)	12 (22.6)	11 (20.8)	24 (49.0)	12 (24.5)	0.435
Thrombocytopenia	6 (5.8)	3 (2.9)	3 (5.7)	1 (1.9)	17 (34.7)	1 (2.0)	0.116
Anemia	9 (8.7)	4 (3.8)	7 (13.2)	11 (20.8)	10 (20.4)	6 (12.2)	0.190
Hepatotoxicity	5 (4.8)	0	4 (7.5)	0	5 (10.2)	0	0.931
Thrombosis	0	1 (1.0)	0	0	0	0	0.777
Lower extremity lymphedema	0	4 (3.8)	0	5 (9.4)	0	0	
Bowel obstruction	0	3 (2.9)	0	1 (1.9)	0	0	
Radiation-induced rectitis	0	0	0	0	0	4 (8.2)	
Radiation induced cystitis	0	0	0	0	0	0	
Femoral head necrosis	0	0	0	0	0	1 (2.0)	

**Table 6:** LOS and hospitalization costs.

	PST (*n *=* *104)	NAC + RS (*n *=* *53)	CCRT (*n *=* *49)	*P*
Hospitalization cost ($)	7969.77 ± 3115.07	9915.97 ± 2553.34	7646.62 ± 2872.94	<0.001^a^
LOS (days)	49.72 ± 19.40	63.08 ± 15.26	54.22 ± 8.50	<0.001^b^

^a^Significant difference between NAC + RS and PST (*P* < 0.001) and NAC + RS and CCRT (*P* < 0.001). No significant difference between PST and CCRT (*P*=0.497). ^b^Significant difference between NAC + RS and PST (*P* < 0.001). No significant difference between NAC + RS and CCRT (*P*=0.07) and PST and CCRT (*P*=0.114).

## Data Availability

The data used to support the findings of this study were supplied by Yongzhang under license and so cannot be made freely available. Requests for access to these data should be made to Yongzhang, zhangyonggx@163.com.
